# A multiclass CNN cascade model for the clinical detection support of cardiac arrhythmia based on subject-exclusive ECG dataset

**DOI:** 10.1007/s13534-022-00246-8

**Published:** 2022-09-12

**Authors:** Carmine Liotto, Alberto Petrillo, Stefania Santini, Gianluca Toscano, Vincenza Tufano

**Affiliations:** 1 Department of Electrical Engineering and Information Technology (DIETI), University of Naples Federico II, 80125 Naples, Italy; 2 Teoresi Group S.p.a., Via Ferrante Imparato, 198, 80146 Naples, Italy

**Keywords:** Cardiovascular disorders, ECG, Arrhythmia classification, Convolutional neural network (CNN), Multiclass CNN cascade, Subject-exclusive criteria

## Abstract

The accurate analysis of Electrocardiogram waveform plays a crucial role for supporting cardiologist in detecting and diagnosing the heartbeat disorders. To improve their detection accuracy, this work is devoted to the design of a novel classification algorithm which is composed of a cascade of two convolutional neural network (CNN), i.e a Binary CNN allowing the detection of the arrhythmic heartbeat and a Multiclass CNN able to recognize the specific disorder. Moreover, by combining the cascade architecture solution with a rule-based data splitting, which leverages the *subject-exclusive* and *balances among the classes* criteria, it is possible predicting the health status of unseen patients. Numerical results, carried out considering Massachusetts Institute of Technology-Beth Israel Hospital arrhythmia database, disclose a classification accuracy of $96.2\%$. Finally, a cross-database performance evaluation and a comparison analysis w.r.t. the current state-of-art further disclose the effectiveness and the efficiency of the proposed solution, as well as its benefits in terms of patient health status prediction.

## Introduction

Cardiovascular disorders are among the most common diseases that seriously threaten human health, especially the middle-aged and older people. They are characterized by high prevalence, high disability, and high mortality. Nowadays, since the world is facing with the aging population, the increasing aggravation of cardiovascular diseases has become a major public health problem [20]. For their evaluation, Electrocardiogram (ECG) analysis is the most effective solution. The ECG is a visual time series which records the electrical activity generated by each cardiac cycle in real-time and it is now widely used in heart rate detection [12]. This non-invasive detection method is easy to operate and has become an essential tool for assisting clinicians in analyzing pathology. At this stage, the judgment of cardiovascular diseases mainly depends on human experience, but, however, there are many types of disorders, and long-term manual detection makes it easy to cause false detection. In addition, the traits of ECG signals include random, low-frequency, and susceptible samples, hence resulting in unstable diagnosis [17]. Therefore, the proper identification/classification of ECG signals plays a crucial role for the treatment of cardiovascular diseases or for an early prevention of them. During last years, the topic has attracted an increasing considerable attention [11, 31] and how to quickly and accurately analyze specific heart diseases has become a new challenging problem [3]. To this aim, the intelligent automatic classification of ECG signals has become an inevitable choice to improve the efficiency and accuracy of ECG recognition [6, 18, 21].

Along this line, machine learning and deep learning tools have been widely leveraged for the assisted diagnosis of heart disease based on ECG signals [1, 9, 15, 29, 30, 33]. Among all the various Deep Learning models, convolutional neural networks (CNNs) architectures have attracted special interest in the field of ECG signal classification and have been successfully applied for the classification of arrhythmias [15]. The main idea behind of using CNN architectures is to extract the meaningful features from the processed data by using a series of spatial convolutions with different filters. Indeed, compared with traditional neural networks, CNNs can automatically extract features, recognize intricate data patterns, and eliminate complex signal pre-processing phases [8]. In this context, for example, [33] implements a simple 1-D CNN consisting of three convolutional layers, three pooling layers, one MultiLayer Perceptron (MLP) layer and one softmax layer. Herein, the ECG beat classification is performed in three main stages: (a) ECG beat detection, (b) samples extraction, (c) classification. Conversely, [1] develops a binary classification model which consists of four convolutional layers - composed of $550 \times 3$, $252 \times 3$, $116 \times 10$ and $50 \times 1$ neurons, respectively- four pooling layers and three MLP layers. To improve CNN performance in recognizing 17 types of heartbeats, [30] introduces batch normalization layers in the proposed 16-layer deep CNN. The proposed model consists of seven convolutional layers, four pooling layers, two batch normalization layers and two dense layers along with softmax layer of 17 neurons. Again, for the classification of 8 classes of heartbeats, [9] suggests an effective long short-term memory (LSTM) recurrent network model while [15] proposes an approach combining the 1D-CNN model and the Stationary Wavelet Transform (SWT) to simultaneously extract features from different wavelet subbands and from the raw ECG signal. The extracted features are then merged and sent to three dense layers, one dropout layer and one softmax. Finally, [29] presents a novel classification technique which combines the feature extraction mechanism of a CNN with the classification method of a radial basis probability neural network (RBPNN). The resulting CNN-RBPNN consists of a signal input layer, signal feature parallel extraction, integration units and an RBPNN classifier.

However, most of the aforementioned works disclose poor classification performance when the classifier evaluates the health status of a new patient, due to the fact that the proposed classification algorithms are patient-specific. Indeed, the ECG waveform may show dissimilarity in morphological and temporal characteristics on the basis of ECG patterns for different patients; therefore the ECG waveforms may be similar to those of the same patient but different respect those of another one.

To overcome this limitation, this work designs a novel classification algorithm which, by leveraging a subject-exclusive training phase, is able to detect, with a high accuracy, the heartbeat disorders of any new patient to be screened, hence endowing our solution with the ability to make correct health prediction for unseen subjects. To this aim, we split ECG tracks in training, validation and test set by taking into account two factors: subject ID and the occurrences distribution of the appraised heartbeat disorder classes. Note that, in order to balance the classes between the three subsets, while keeping the model subject-exclusive, we exploit a rule-based methodology. Based on these data set, we propose a classification model composed of a cascade of binary and a multiclass CNNs whose combined action allows improving the accuracy results. Indeed, the proposed solution improves the accuracy achievable when using the solely multiclass CNN model of about $14.1\%$, hence reaching a final F1 score of $96.3\%$.

Finally, the paper is organized as follows. Section 2 presents the methods and the materials leveraged in this work. The proposed novel classification algorithm, based on the cascade of two CNNs, are detailed in Sect. 3. Section 4 discloses the obtained experimental results and the effectiveness of the proposed approach w.r.t. the technical literature. Conclusions are drawn in Sect. 5.

## Methods and materials

### ECG data set

In order to training and evaluate the effectiveness of the proposed ECG classification model we consider the well-known public MIT-BIH Arrhythmia database (MITDB) [22]. It contains 48 ECG records from 47 patients, each of which is sampled at 360 [Hz] with a duration of 1800 [s] and 11 bit resolution per sample. The cohort of 47 subjects is composed of 25 male patients aged 32–89 years and 22 female patients aged 23–89 years. Among the 48 ECG records, 23, enumerated from 100 to 124 into the database, are randomly extracted from a collection of over 4000 Holter tapes (of 24 hours of duration) and include the clinical routine arrhythmias. The remaining 25 records, enumerated from 200 to 243, are selected to include significant arrhythmias which cannot be detected with small random samples, such as the ventricular, junctional, supraventricular ones and cardiac conduction abnormalities. The records are obtained by placing electrodes on the patient chest via two channel, i.e. a Modified Limb lead II (MLII) in the majority of cases and a modified V1 (occasionally V2/V5 and V4 in a single case). Note that the data extraction is carried out by two experts cardiologists. Each record is accompanied with an annotation file, where the R peaks time occurrence and the corresponding class for each ECG beat is highlighted, as well as all the multiple ECG morphologies. In this work we focus on four kind of ECG beats, namely: (1) Normal beats (N); (2) premature ventricular contraction (PVC); (3) left bundle branch block (LBBB); (4) right bundle branch block (RBBB).

### Data pre-processing

**Fig. 1:**
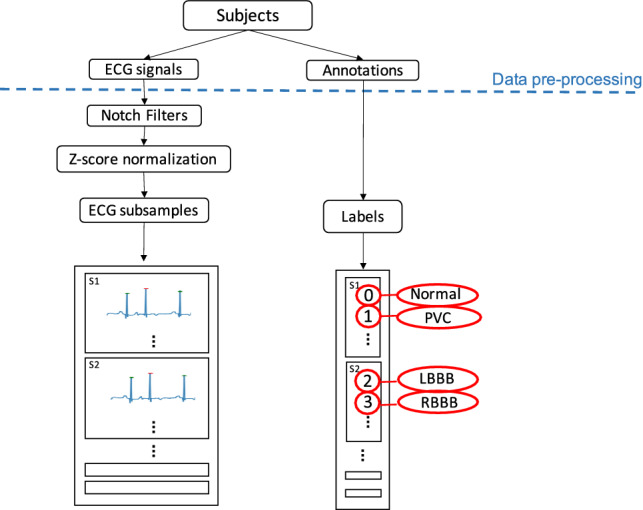
Schematic overview of the data pre-processing

The aim of this phase is to process the ECG dataset, as presented in Sect. 2.1, and to select the proper data features so to obtain a customized dataset to be processed by the proposed classification algorithm. Specifically, the data pre-processing stage consists of three different phases as described in what follows and schematized in Fig. 1.

#### Noise filtering and normalization

In order to remove noise measurements from ECG signal, due to the well-known phenomena of electromagnetic distortion related to ECG instrument, we exploit two Notch filters [7]. The first one, with a cut-off frequency of 50 [Hz], aims at suppressing the power-line interference while the second one, characterized by a cut-off frequency of 0.05 [Hz], is used to remove the baseline noise.

Finally, to avoid improperly weighting the ECG signals, a data normalization operation is carried out via the Z-score technique, widely used in machine learning field [23].

#### ECG data selection

At this pre-processing stage, we choose, from the MIT-BIH Arrhythmia database (MITDB), the signals containing certain types of annotated cardiac beats while ignoring any non-beat annotations. To this aim, by leveraging the annotation file, we perform the data selection considering only the ECG signals characterized by the following annotations classes:

- N
- PVC
- LBBB
- RBBB

#### Customized dataset generation and labelling

**Fig. 2:**
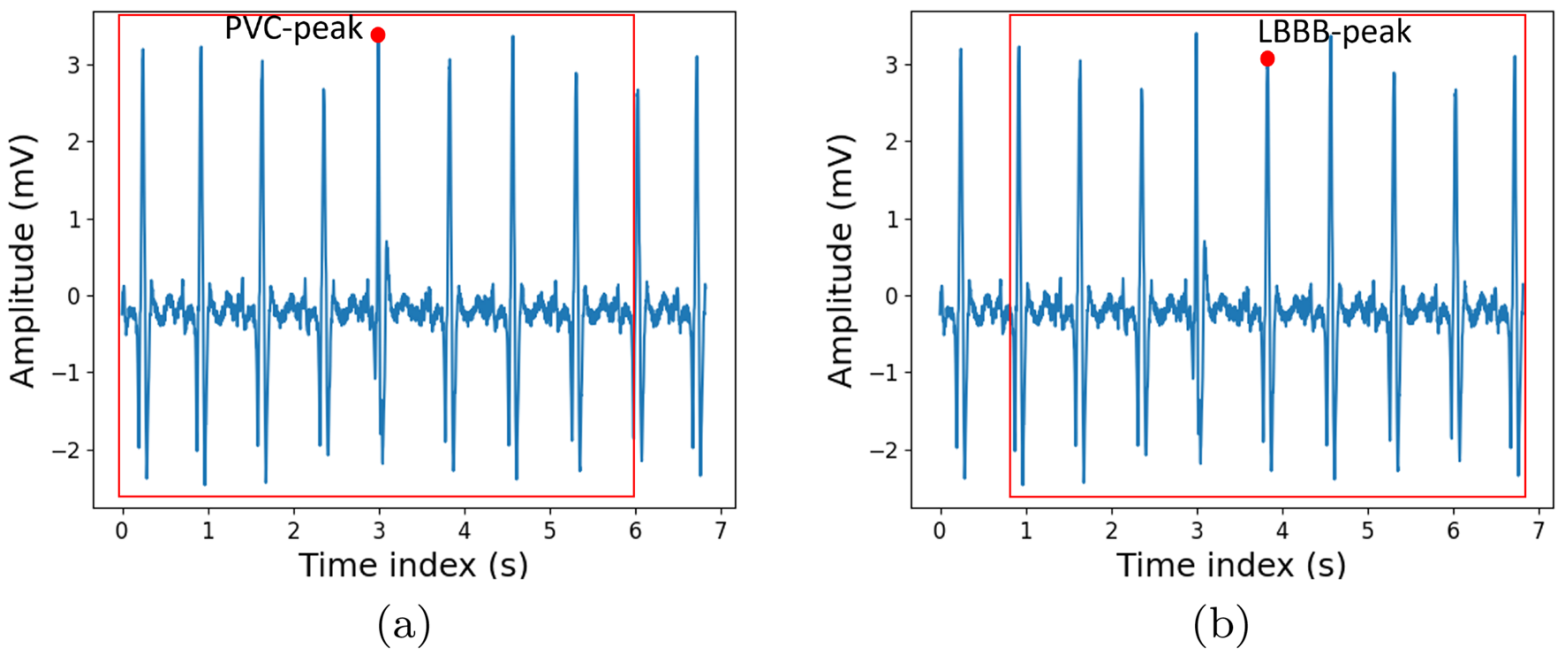
Exemplary samples of the extracted ECG sections

With the aim of enhancing the training process and the identification capability of the proposed classification algorithm, we construct a customized dataset which is composed of a number of ECG signals larger than the original one. Specifically, each ECG signal selected in Sect. 2.2.2, whose duration is about 30 min, is divided into different sections, of a duration of 6 [s], centered around each R peak present into the ECG signal. Bearing in mind that the ECGs included in the original dataset are acquired with a sampling frequency of 360 [Hz], each extracted section comprises 2160 data points. These sections are identified by considering all the R peaks within the appraised ECG signal. The R peaks are isolated by considering the time index referring to the annotated beat. For each of these peaks we create a new time series by considering the first 3 [s] before and after the time this peak occurs. Examples of two subsequent ECG samples are shown in Fig. 2.

In so doing, we obtain a new customized dataset which allows improving the classification algorithm performance since this latter is not only able to learn and classify the specific R peak, but also to recognize it through the ECG trend bringing to the peak itself.

Then, each section is labelled with an enumerative identifier which specifies the typology of the occurring peak. Note that, for the labelling process of each section, the annotations referred to eventual peaks present in the 3 [s] before and after the central significant peak are neglected. Therefore the final data-frame, on which the classification algorithm works, contains 36,950 ECG samples, each of them labeled as: 0 if the section present a *N* peak; 1 if the peak is PVC; 2 when the peak is *LBBB*; 3 in the case of *RBBB* peak.

**Table 1:** CNN model architecture

# Layers	Type	Kernel size	Stride	Kernel	%
2	Conv 1D	5	1	16	–
1	Max pooling	2	1	–	–
1	Dropout	–	–	–	10
2	Conv 1D	3	1	32	–
1	Max pooling	2	2	–	–
1	Dropout	–	–	–	10
2	Conv 1D	3	1	32	–
1	Max pooling	2	1	–	–
1	Dropout	–	–	–	10
2	Conv 1D	3	1	256	–
1	Global max pooling	2	1	–	–
1	Dropout	–	–	–	20
1	Dense	–	–	64	–
1	Dense	–	–	64	–
1	Dense	–	–	4	–

**Table 2:** Differences between binary and multiclass CNN

	Softmax neurons	Loss function	Epochs
Binary CNN	2	Binary cross-entropy	10
Multiclass CNN	3	Sparse categorical cross-entropy	18

**Fig. 3:**
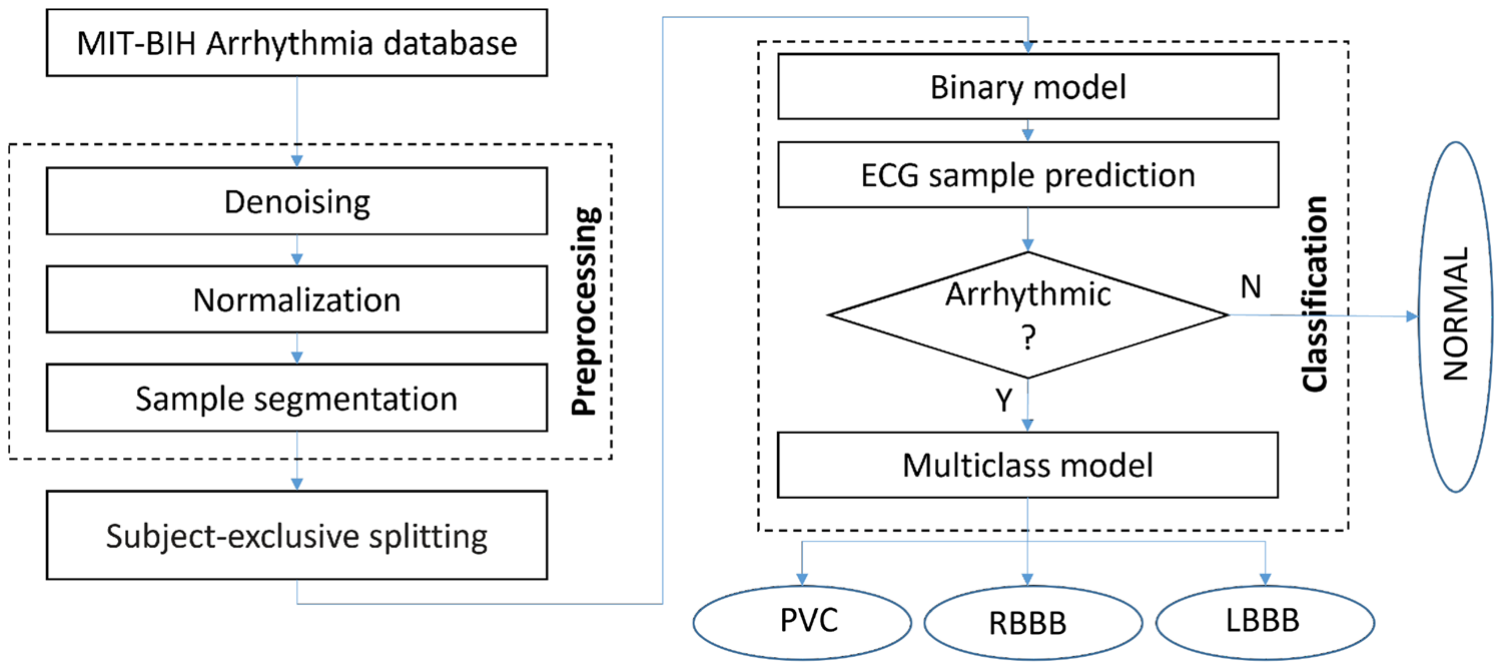
Schematic overview of the proposed classification methodology

## Proposed methodology

### Multiclass CNN cascade

This section describes the proposed ECG heartbeats classification algorithm and details all the tools we leveraged for its design. Specifically, it is designed via a cascade of CNNs composed of: (1) a Binary CNN, allowing the detection of the arrhythmic heartbeat from ECG tracks and their isolation from the non-arrhythmic one; (2) a Multiclass CNN which, based on the output of the Binary CNN, is able to detect the specific heart rhythm disorder, i.e. LBBB, RBBB, PVC.

Figure 3 shows an overview of the classification algorithm. Since the processed ECG recordings are one dimensional time series, we exploit 1-D CNN layers type to develop both the Binary CNN and the Multiclass one for an effective heartbeats classification. Each CNN encompasses 19 layers, namely: 8 Convolution1D (Conv1D) layers; 3 Max Pooling layers; 1 Global Max Pooling; 4 Dropout layers; 3 Dense layers. The main features of each layer are summarized in Table 1.

In addition to them, the Binary CNN is characterized by a Softmax final layer with 2 neurons while this latter is composed of 3 neurons for the Multiclass one. See Table 2 for details about their features.

### Binary CNN

The binary CNN receives as input 1D time series ECG tracks and provides as output a first possible indication about the heartbeats disorder, i.e. if the signal is arrhythmic or not. Each cardiac track is put in input to a layer of size $2160 \times 1$ and then it is processed by a combination of multiple convolution, pooling and dropout layers. The first of these layers aims at extracting the features of the ECG wave by using a series of 1-dimensional convolutions. Each layer leverages multiple 1-D kernels and is characterized by neurons with a rectified linear unit (ReLu) activation function (see [2] for detail about the activation function). In so doing, the output feature map is provided. In order to retain the significant extractable data features, the pooling layer with MaxPooling technique [26] is exploited. This allows reducing the number of non-significant extracted features, as well as the computational complexity of the learning process for the CNN. The subsequent dropout layer is used for preventing the overfitting of the neural network. Then, this kind of process is repeated 3 times. After this data processing, we design three Dense layers (whose neurons activate according to the ReLu function), well known in the technical literature as Fully Connected layers [13], so to concatenate the convolution/pooling outputs data into a single features vector. Finally, the last layer of the binary CNN is a Dense Layer characterized by 2 neurons with a Softmax activation function ( see [4] for detail about the activation function). It performs the classification stage by optimizing the CNN model parameters through the minimization of a loss function, i.e. the Binary Cross-Entropy function defined as

1 $$\begin{aligned} Loss = \frac{-1}{n}\sum _{c=1}^N\big (\big [y_c*log(a_c)+(1-y_c)log(1-a_c)\big ]\big ) \end{aligned}$$

where $y_c$ is the target value; *c* is the class index; $N=2$ is the total number of classes; *a* is the true value. Note that, $y_c$ and *a* are one-hot encoded. The choice of selecting this kind of loss function is made because of the binary nature of the classification problem (see details in [24]). In this way, the Binary CNN provides the estimated probability for each ECG track in belonging to the class *Normal* or *Arrhythmic*.

### Multiclass CNN

The Multiclass CNN receives in input the ECG tracks estimated as *Arrhythmic* by the Binary CNN and provides indication about the specific appraised heartbeat disorder, i.e. PVC, RBBB and LBBB. The internal structure of CNN is similar to one of the Binary CNN, except for the final output layer. Specifically, this latter is designed such that three neurons, with Softmax activation function, provide the estimated ECG arrhythmic peaks by optimizing a Sparse Categorical Cross-Entropy loss function (see details in [19]), defined as (1) with $N=3$ and where $y_c$ and *a* are not one-hot encoded.

### Learning algorithm

The training phase of each model is performed so to prevent the phenomenon of the overfitting. The dataset is split w.r.t. a ”subject-exclusive” criteria (see the next ”Experimental results” section) in training, validation and test sets. Therefore, the ECG segments for training set are shuffled with a random seed with the aim of reducing variance and making sure that models remain general and less overfitted. Actually, this prevents any bias during training and the model does not learn the order of the training set.

The networks are trained via the stochastic gradient descent method and the back propagation (BP) algorithm by using the ADAM44 [16] (ADAptive Moment estimation) optimizer with a default learning rate of $0.1\%$. During the training process, the batch size is set to 32 examples over 50 training epochs. However, the proposed architecture rapidly converges and reaches a stable training and testing performance after 10–18 epochs approximately.

Three techniques are adopted to mitigate the overfitting risk, namely Dropout, Early stopping and Model checkpoint.

#### Dropout

The dropout mechanism is implemented through the above-mentioned dropout layers. Specifically, this method [27], by randomly removing a number of connections between some nodes of the deep neural networks, consists in dropping units from the network during training. In this way, we exclude the development of a codependency among neural units and we force the network to be accurate, even in absence of certain information. We add a dropout layer after each Max Pooling layer with a dropout rate of 10%, and a dropout layer with a dropout rate of 20% after the Global Max Pooling Layer as reported in Table 1.

#### Early stopping

Early Stopping is used to avoid overfitting. This technique interrupts the training stage when a monitored measure, for example the validation error, has not improved for some amount of time [5]. As monitoring value, we choose the accuracy metric evaluated on validation data, and, as condition to stop the training, we choose the invariance of this metric for a number of 10 consecutive epochs.

#### Model checkpoint

The model Checkpoint option, provided by Keras [14], automatically saves the weights of the best suitable model in terms of a specified metric [25]. We choose to monitor the accuracy value evaluated on validation data so to keep the weights of the model with the higher accuracy.

## Experimental results and analysis

**Fig. 4:**
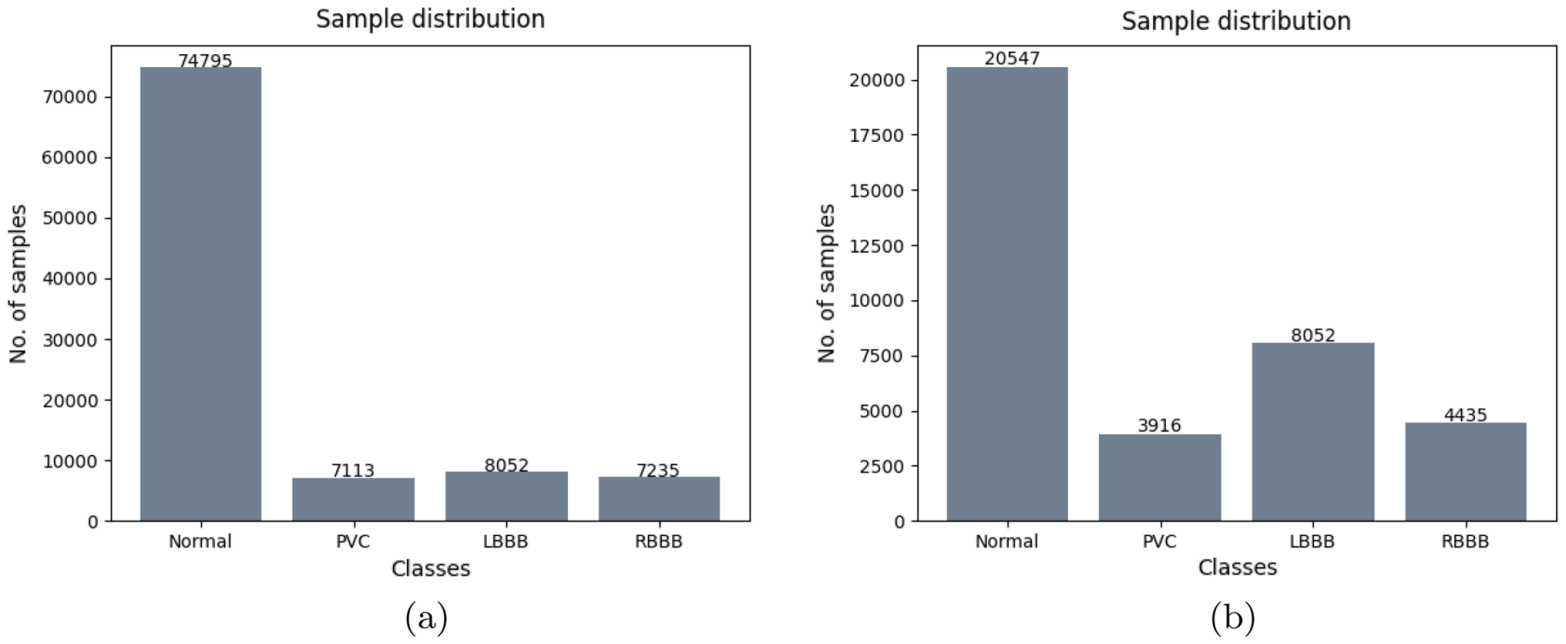
Sample distribution of the ECG beats after the pre-processing of the MIT-BIH dataset: **a** Initial distribution; **b** Balanced distribution

**Fig. 5:**
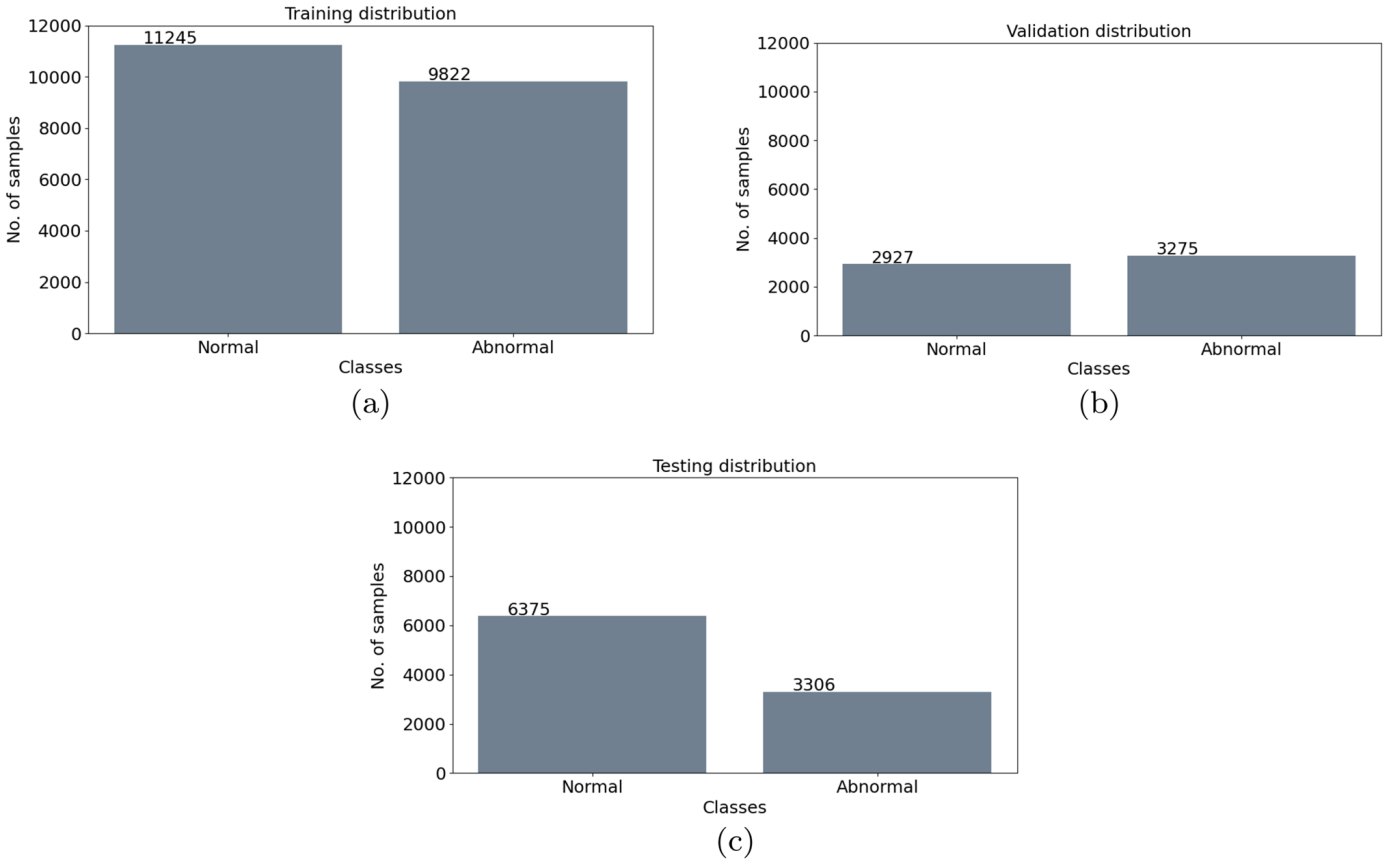
Subdivision of the samples distribution for the Binary CNN into: **a** Training set, **b** validation set, **c** test set

### Experimental configuration

The customized dataset, derived in Sect. 2.2.3, is divided into three groups, namely: (1) Training set; (2) Validation set; (3) Test set. Given the large discrepancy of the data distribution into the different appraised classes, due to the presence of a higher number of *Normal* ECG signals (see Fig. 4a), for the generation of the training set, we have randomly removed some of the signals belonging to the this class. Specifically, this operation is performed by eliminating the same number of *Normal* ECG track, i.e. 17,000 signals. Note that, this guarantees a uniformity into the data class distribution for each patient. Subsequently, all the ECG tracks, related to patients unaffected by arrhythmias or affected by a neglected percentage of them, are removed from both the training and validation sets, i.e. 17 subjects. In so doing, we derive the final dataset for the training of the proposed classification algorithm and for its validation. See Fig. 4b.

More in detail, data subdivision into the appraised three groups, i.e. Training, Validation and Test set, is carried out by taking into account two criteria:

- Subject-exclusive: all the ECG sub-tracks, related to the same patient, are included into the same sub-set (i.e. Training set). This splitting modality guarantees that the system will be not tested or validated on samples belonging to the same patient on which the classification algorithm has been trained. Indeed, different PQRST tracks, belonging to the same patient, are more similar to each other than those belonging to different patients. In this way, we ensure that the information learned by the CNN is independent from the specific patient.
- Balance among the classes: the patients are included in the different sub-datasets by considering also the occurrence of each kind of arrhythmia. For example, if we have three patients with many ECG sub-tracks labeled as PVC we split the patients in each of the three sub-set. In so doing, we ensure the balance among the sub-dataset. This is crucial for an improved functioning of the classification algorithm [28].

**Fig. 6:**
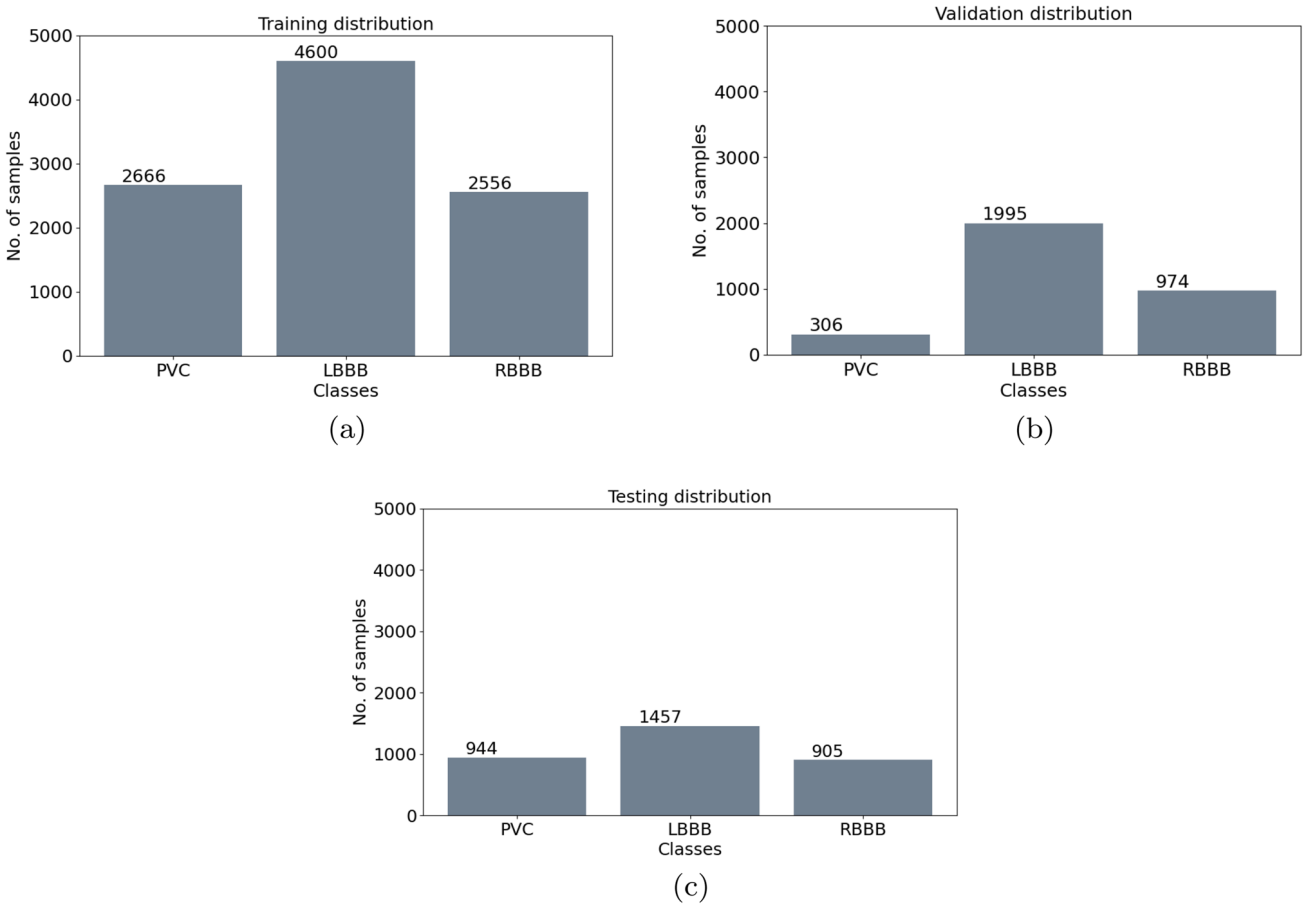
Subdivision of the samples distribution for the Multiclass CNN into: **a** Training set, **b** validation set, **c** test set

According to the above-mentioned criteria, data are finally subdivided as in Fig. 5 for the Binary CNN and as in Fig. 6 for Multiclass CNN.

### Implementation

The architectures of the binary and multiclass models are separately defined and sequentially invoked. From the implementation point of view, two most important phases are performed: the training and the testing phase, both run in batches. During the training phase, the two models (i.e. the Binary and Multiclass CNNs) are separately trained. The Binary CNN is trained and validated on the Training and Validation set considering only two classes: *Normal* and *Abnormal*. The latter one is obtained by aggregating all the arrhythmic ECG tracks. The distribution of the data for the training and validation set is shown in Fig. 5a, b. The Multiclass CNN is trained considering only the three abnormal classes, whose distribution is shown in Fig. 6a, b. Afterwards, the two models are queried in cascade. First of all, the binary model is initialized and queried by using the data shown in Fig. 5c (i.e. the Test set) in order to obtain the binary prediction. Then, the multiclass model is initialized and queried with only the *Abnormal* predicted data for the multiclass prediction.

The experiments are performed by using a processor Intel ^®^Core™ i7-10510U 1,8 GHz, 8 GB 2666 MT DDR4 of memory, GPU NVIDIA ^®^ GeForce ^®^ MX130 (with 2 GB GDDR5 of memory) and Windows 10 Home operating system. For the design of the neural networks, we exploit the Python programming language and, more specifically, the following well-known open sources software libraries, namely: (1) Tensor Flow, realized by Google and dedicated to the automatic learning; (2) Keras, a API, supported by Tensor Flow and specifically designed for the training and the validation of the neural networks; (3) Pandas, for the storage and management of the ECG tracks; (4) wfdb, for loading the ECG records and annotation files; (5) HeartPy, for filtering and manipulating the ECG signals. Moreover, the whole software architecture is implemented in the integrated development environment of *PyCharm* which allows creating a convenient environment for productive Python and data science development.

### Key performances indexes

**Fig. 7:**
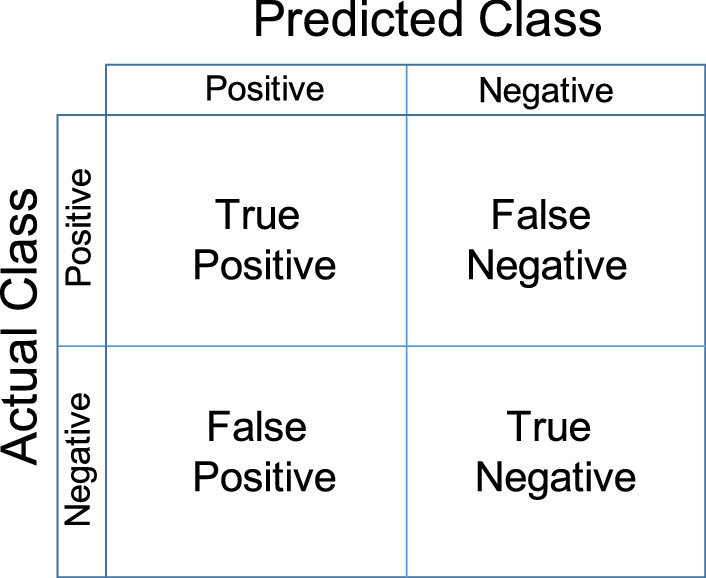
Confusion matrix structure

The performance of the proposed Multiclass CNN Cascade is evaluated by leveraging four statistical metrics: (1) Precision; (2) Recall; (3) Accuracy; (4) F1 score. These statistical key performances indexes (KPIs) are computed as follows:

2 $$\begin{aligned} \mathrm {Precision }&= {\frac{Tp}{Tp + Fp}} \end{aligned}$$

3 $$\begin{aligned} \mathrm {Recall}&=  {\frac{Tp}{Tp + Fn}} \end{aligned}$$

4 $$\begin{aligned} \mathrm {Accuracy}&=  {\frac{Tp + Tn}{Tp + Tn + Fp + Fn}} \end{aligned}$$

5 $$\begin{aligned} \mathrm {F1 }&=  {2\frac{Precision*Recall}{Precision + Recall}} \end{aligned}$$

where *Tp* denotes the number of cases rightly classified as positive; *Fp* represents the number of cases wrongly classified as positive; *Tn* denotes the number of cases rightly classified as not positive; *Fn* represents the number of cases wrongly classified as not positive.

In addition, classification results can be also visualized via the confusion matrix (see an exemplary structure in Fig. 7), which shows the *True Positive*/*True Negative* on the main diagonal and the *False Positive*/*False Negative* on the secondary diagonal.

### Results discussions

**Fig. 8:**
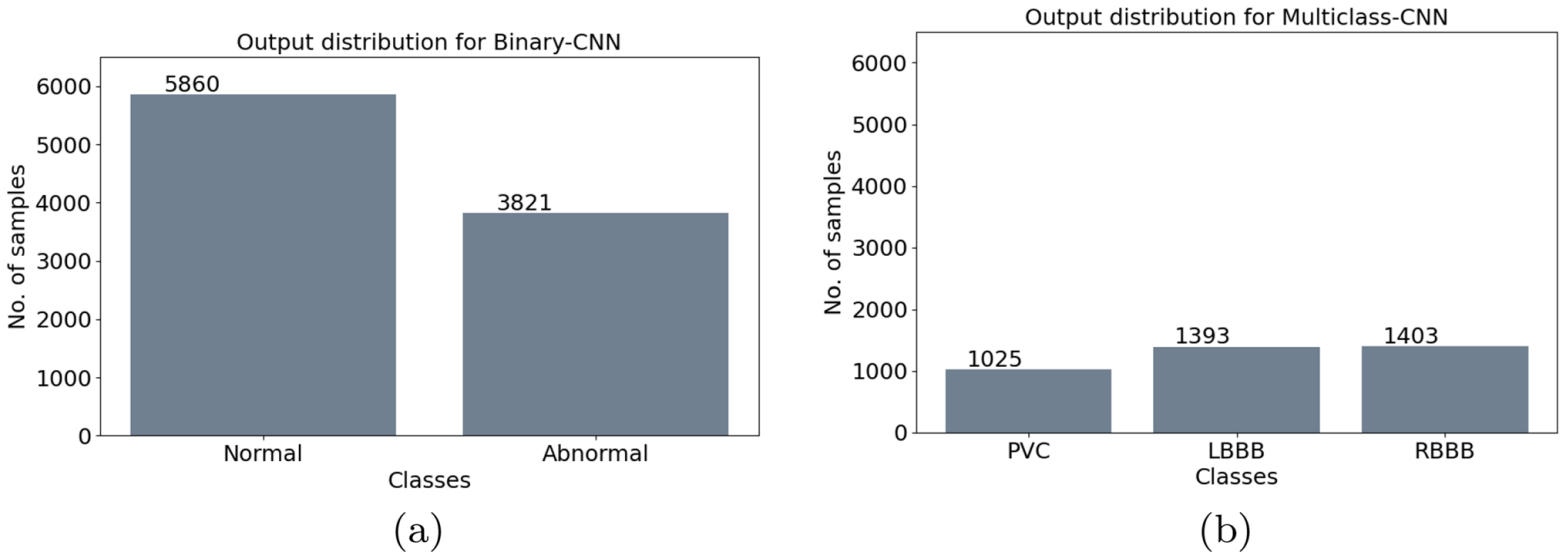
Data output distribution for: **a** Binary CNN, **b** multiclass CNN

The proposed Multiclass CNN Cascade diagnoses and classifies four different types of arrhythmias, i.e., N, PVC, LBBB and RBBB with a final F1 score of about $96.2\%$. The Binary CNN is tested on the data shown in Fig. 5c. Among the data, the one classified as ”abnormal”, reported in Fig. 8a, are subsequently put in input to the Multiclass CNN, which predicts the disorders distribution as in Fig. 8b.

**Fig. 9:**
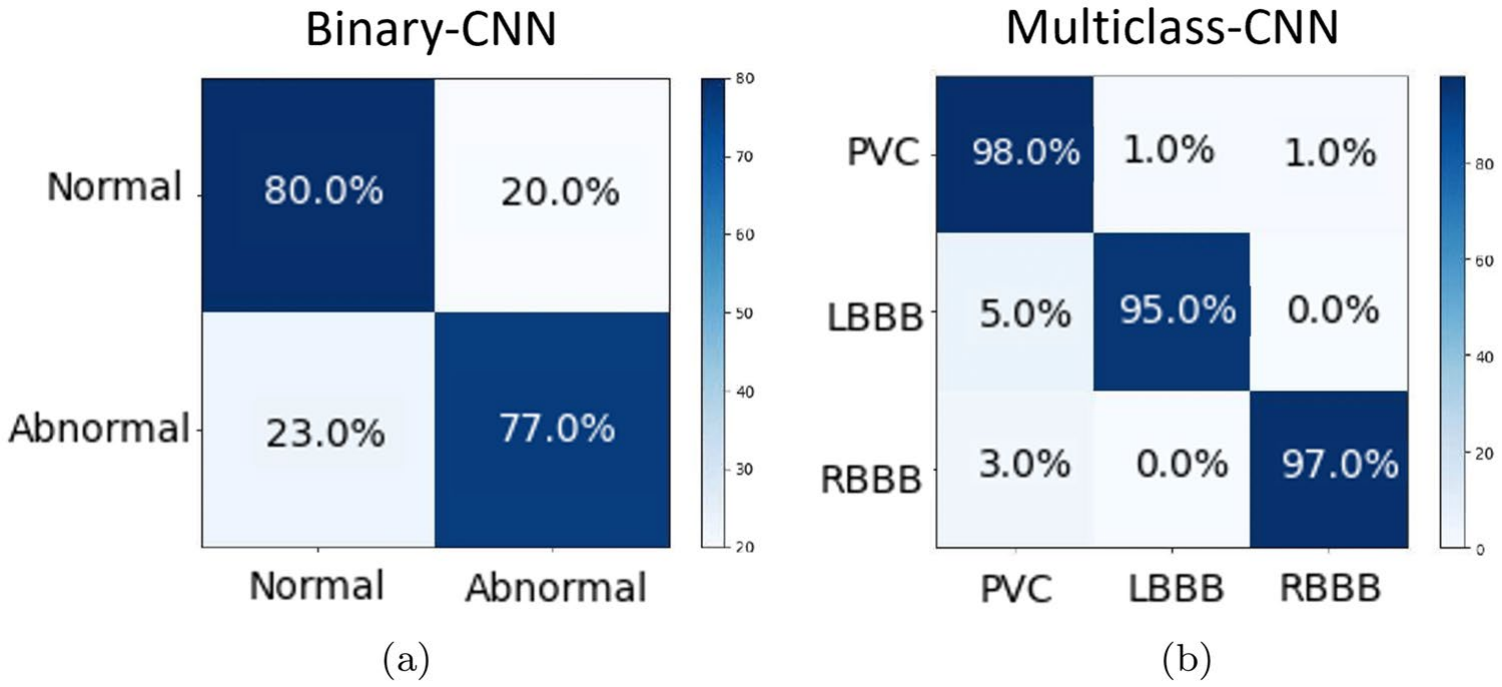
Performance analysis. Confusion matrix for: **a** Binary CNN model, **b** multiclass CNN model

A detailed performance description of the proposed CNN Cascade is carried out via the analysis of the confusion matrices whose diagonal elements show the correctly classified classes, whereas the off-diagonal elements represent an incorrect classification. Confusion matrix for the Binary CNN is reported in Fig. 9a while the Multiclass CNN is depicted in Fig. 9b. As it is possible to observe in Fig. 9a, the proposed Binary CNN is able to correctly recognize, in $80 \%$ of the cases, the Normal heartbeats and, in $77 \%$ of the cases, the abnormal ones with a total accuracy of about $78.5 \%$. These percentage values are obtained by querying the Binary CNN with 8 new subjects, for a total of 9681 6-seconds ECG tracks, composed of 6375 labeled as *Normal* and 3306 labeled as *Abnormal*. The $80\%$ of the first class is correctly predicted as *Normal* with the $20\%$ of false positive. For the other class, the $77\%$ is correctly predicted as *Abnormal* with the $23\%$ of false negative. The predicted distribution is shown in Fig. 8a. The classification ability is improved by the Multiclass CNN, as observable in the Fig. 9b, where it is shown an almost diagonal confusion matrix. As highlighted herein, the Multiclass CNN correctly detects the PVC disorders in $98\%$ of the cases, the LBBB ones in $95\%$ of the cases and the RBBB ones in $98\%$ of the cases. In this case, the total obtained accuracy is about $96.2\%$ due to the pre-classification benefits provided by Binary CNN. Indeed, by excluding the ”Normal” samples from the second classifier, it is possible to train the second CNN model on the basis of a more balanced set of ”Abnormal” samples heartbeats. Furthermore, the value of the KPIs (in Sect. 4.3) for the CNN Cascade are reported in Table 3. Herein, we also compare the performance of the cascade with the one achievable via a unique Multiclass CNN which also predicts the Normal heartbeat activity. Comparison results (reported in Table 3) clearly underline the effectiveness and the benefits which the cascade solution could bring in terms of prediction ability.

Finally, with the aim at better disclosing the advantages of the proposed Multiclass CNN cascade solution, we also perform a comparison w.r.t. other approaches suggested in the technical literature, again by leveraging the KPIs in Sect. 4.3 and by also considering the subject-exclusive criteria. Note that, this latter criteria is crucial for an effective classification algorithm design since it ensures that the information learned by the CNN is independent from the specific patient. Comparison results, reported in Table 4, highlight how, if we restrict our attention to the methods which are subject-exclusive, the proposed cascade solution allows obtaining the improved results in terms of accuracy and *F*1 score.

**Table 3:** Evaluation metrics results

	Multiclass CNN (%)	Multiclass CNN cascade (%)
Accuracy	82.1	96.2
Precision	89.2	96.4
Recall	81.8	96.2
F1 score	84.4	96.3

Effectiveness and benefits of the proposed solution compared to a unique Multiclass CNN also predicting the N heartbeat

**Table 4:** Accuracy comparison between works

Works	Accuracy (%)	F1 score	Recall	Precision	MIT-BIH DB	Subject exclusive
[15]	99.6	–	–	–	Yes	No
[33]	92.7	–	–	–	Yes	Yes
[1]	93.53	–	–	—	No	No
[30]	95.2	–	–	–	Yes	No
[9]	98.7	98.4%	98.7%	98%	Yes	No
[29]	86.2	86%	85.4%	–	No	No
Proposed solution	96.2	97.1%	96.5%	94.3%	Yes	Yes

### Cross-database performance evaluation

In order to validate the generalization ability of our Multiclass CNN Cascade in predicting and classifying heartbeat disorders, herein we perform a cross-database evaluation by using two alternative ECG database, namely: (1) MIT-BIH Long Term [10]; (2) Shaoxing and Nyngbo [32]. For a fair comparison analysis, the ECG signals of both the databases are pre-proccessed according to the methodology exposed in Sect. 2, i.e. they are re-sampled at 360 [Hz] using linear interpolation and, then, each of them, divided into sections of 6 [s] duration.

MIT-BIH Long Term contains 7 long-term ECG recordings (of about 14–22 h) sampled at 128 [Hz], with manually reviewed beat annotations. Note that, in this database, the annotations only include *Normal* and *PVC* classes and, hence, the classification ability is evaluated w.r.t. them. The performance of the proposed Multiclass CNN Cascade on this database can be summarized as follows: a classification accuracy of about 85$\%$ is achieved for arrhythmic ECG signals while, among the correctly classified arrhythmic samples, the 99$\%$ are correctly recognized as belonging to *PVC* class.

The Shaoxing and Nyngbo ECG Database is a research database for 12-lead electrocardiogram signals which is created under the auspices of Chapman University and Shaoxing People’s Hospital (Shaoxing Hospital Zhejiang University School of Medicine). Database contains 12-lead ECGs of 10,646 patients, with a sampling rate 500 [Hz], and features 11 common rhythms and 67 additional cardiovascular conditions, all of them labeled by professional experts. The dataset is characterized by 10 [s], 12-dimension ECG signals describing a widely range of heartbeat disorders, including the ones able to be classified by our Multiclass CNN cascade. The performance of the proposed solution on this database, evaluated only w.r.t. the four classes appraised in this work, can be summarized as follow. The overall classification accuracy is about 80$\%$ with an F1-score equal to 79.8$\%$ for arrhythmic ECG signals. Note that, although the obtained performance reveals a good classification and prediction ability, it is worth noting that accuracy and F1 score values are lower than the ones guaranteed on MIT-BIH due to the different acquisition method used for constructing the Shaoxing and Nyngbo ECG Database.

## Conclusion

Improving the accuracy of the heartbeat disorders classification is a crucial task for the prevention and diagnosis of cardiac pathologies. To this aim, this paper is devoted to propose a novel CNN-based architecture, composed of two CNN in cascade, for their accurate detection. The proposed multiclass CNN model is designed such that it enhances the prediction accuracy in different way, namely: (1) the model is endowed with an ability to make correct health prediction for unseen subjects; (2) the cascade solution allows making a more accurate prediction of the ”Abnormal” tracks. Moreover, it is worthy underline how the proposed methodology is embedded with a specific rule-based data splitting which helps maintaining the balance among the classes in training, validation and test set while remaining the dataset subject-exclusive. Numerical results highlight these aspects and Table 3 shows an outstanding performance with a classification accuracy of $96.2\%$ for heartbeat disorders. A possible drawback of the proposed solution is related to the data splitting phase which requires a deep analysis of the data and a lots of parameters to take into account. Future works in this direction could include the implementation of an automatic rule-based algorithm for this multi-objective optimization problem, hence further improving the balancing of the three subsets.
